# Food Allergy Symptom Self-Management With Technology (FASST) mHealth Intervention to Address Psychosocial Outcomes in Caregivers of Children With Newly Diagnosed Food Allergy: Protocol for a Pilot Randomized Controlled Trial

**DOI:** 10.2196/25805

**Published:** 2021-03-03

**Authors:** Brantlee Broome, Mohan Madisetti, Margaret Prentice, Kelli Wong Williams, Teresa Kelechi

**Affiliations:** 1 College of Nursing Medical University of South Carolina Charleston, SC United States; 2 College of Medicine Medical University of South Carolina Charleston, SC United States

**Keywords:** caregiver well-being, food allergy, self-management, mhealth, randomizes mixed trial, caregiver, well-being, emergency room, smartphone app, smartphone, children

## Abstract

**Background:**

Approximately 2.4 million children in the United States suffer from food-induced anaphylaxis, a condition that is annually responsible for over 200 deaths and 200,000 emergency room visits. As a result, caregivers of children newly diagnosed with severe and life-threatening food allergic reactions experience clinically significant symptoms of psychological distress, including fatigue, anxiety, depressed mood, social isolation, and substantially reduced quality of life. Despite this recognition, there is a lack of caregiver-centered self-management interventions to address these concerns.

**Objective:**

In this protocol, we propose to develop and conduct feasibility testing of a technology-enhanced, self-management, mobile health, smartphone app intervention called Food Allergy Symptom Self-Management with Technology for Caregivers (FASST) designed to meet the psychosocial health needs of caregivers of children with a new diagnosis of food allergy.

**Methods:**

This pilot study uses qualitative work (Phase I) to inform a 4-week longitudinal randomized controlled trial (Phase II). In Phase I, 10 caregivers of children (≤18 years old) with established food allergy (≥1 year from diagnosis) will participate in semistructured interviews to inform the development of the FASST app. In Phase II, 30 caregivers of children (≤18 years old) with a newly diagnosed food allergy (≤90 days from diagnosis) will be randomized 2:1 to receive the FASST intervention (n=20) or control condition (basic app with educational resources; n=10). Process measures include feasibility, caregiver acceptability, adherence, and satisfaction. Outcome measures include caregiver fatigue, anxiety, depression, sleep, self-efficacy, and quality of life measured at baseline, week 4, and 3 months post study completion.

**Results:**

Phase I study activities have been completed, and Phase II participant enrollment into the randomized controlled trial is expected to commence in 2021.

**Conclusions:**

With limited readily available resources at their disposal, the results from this study have the potential to provide caregivers of children with a newly diagnosed food allergy a tool to help them self-manage and mitigate negative psychosocial factors during a critical time period in the caregiving/condition trajectory.

**Trial Registration:**

ClinicalTrials.gov Identifier NCT04512924: https://clinicaltrials.gov/ct2/show/NCT04512924

**International Registered Report Identifier (IRRID):**

DERR1-10.2196/25805

## Introduction

In the United States, approximately 6 million children suffer from food allergies (FAs) [1], a major public health concern as its prevalence has continued to increase over the past 2 decades [2]. Food-induced anaphylaxis (FIA) is an associated health risk affecting more than 40% of children diagnosed with FA [2]. Although FIA reactions in children are rare, they are responsible for over 200 deaths and 200,000 emergency room visits annually in the United States [3]. There is no cure for FA, and standard of care is strict avoidance of implicated food allergens and emergency medical treatment of symptoms after exposure [3]. There are many challenges to a caregiver’s ability to adhere to this standard of care—most notable is the potential for a fatal reaction from ingestion of food substances that are often invisible due to cross-contact with contaminated surfaces or the presence of unidentifiable food ingredients within processed foods. This can create an ever-present and heightened state of vigilance, anxiety, and stress-related fatigue as caregivers learn to both manage FA as a chronic condition and respond to food-induced reactions as an acute illness or event. Caregivers of children with newly diagnosed FA experience significant life behavior alterations that can test their ability to balance appropriate vigilance and management strategies while tempering the effects on quality of life [4].

### Background

The principal investigator’s (PI’s) research, and that of others, highlights that caregivers of children with FA describe the physical challenges of care as overwhelming and constant. The perpetual hypervigilance and ensuing exhaustion associated with time-consuming and persistent condition-management activities—such as monitoring a child’s food consumption at school or when with friends and when shopping for and preparing special food—have a significant impact on caregiver quality of life and are associated with fatigue, uncertainty, constant stress, social isolation, reduced spontaneity, and persistent anxiety, fear, and depression [5-12]. Following diagnosis, caregivers experience a period of psychosocial adjustment where these symptoms are most pronounced [13]. As caregivers begin to understand the necessary precautions and potential consequences of accidental ingestion associated with FIA and the required condition management activities, fear and anxiety emerge as predominant emotions. While a certain level of anxiety is essential for adequate management, high levels of sustained anxiety in caregivers of children with FA can be maladaptive, increasing the overall burden of caring for a child with FIA and negatively impacting the caregiver’s ability to provide care to self, child, and family [14]. Symptom self-management is exceedingly relevant to the caregiver of a child with newly diagnosed FA(s) as condition management is complex and compounded by factors outside of the caregiver’s control and is further intensified by the lack of definitive treatment or cure.

Our recent preliminary research [5] corroborates findings in the literature [6-14], suggesting that 32% of caregivers with a child who is newly diagnosed with FA(s) experience clinically significant psychological distress [15]. Despite recognition that family caregivers of children with severe FAs experience these adverse psychosocial symptoms and decreased quality of life, there are a lack of caregiver-centered self-management interventions to address their symptoms. The lack of self-management interventions may lead to persistence of these distressing symptoms, possibly beyond the period of initial diagnosis.

In this protocol, we propose a 2-phase pilot study to develop a mobile health (mHealth) smartphone app intervention called Food Allergy Symptom Self-Management with Technology for Caregivers (FASST) that specifically addresses psychosocial symptoms experienced by caregivers of children with newly diagnosed FA by providing targeted education and the ability to self-monitor and self-manage experienced symptoms. In Phase I of the study, we will conduct key informant interviews and collect data from caregivers of children (≤18 years old) with established FA (diagnosed with FA ≥1 year ago) to guide the development process for the FASST app. We consider these caregiver interviewees as co-designers of our intervention as they possess a level of experience and understanding of FA management and the psychosocial consequences of caring for a child during the critical period following a child’s initial FA diagnosis that caregivers of newly diagnosed children do not. In Phase II, and over the course of 6 months, we will conduct preliminary feasibility testing of the FASST app in a 4-week longitudinal, pilot, randomized controlled trial of 30 caregivers of children ≤18 years of age who are newly diagnosed (≤90 days from diagnosis) with FA(s).

### Aims

Using qualititave Phase I work, the primary aim of the main study (Phase II) is to conduct preliminary testing of implementation processes including feasibility, acceptability, adherence, and satisfaction (using the RE-AIM [Reach, Effectiveness, Adoption, Implementation, and Maintenance] framework with process measures, surveys, and key informant interviews) among 30 caregivers randomized to use FASST (n=20) or the control condition (ie, basic app with resource links; n=10) over 4 weeks. The secondary aim of this phase is to obtain estimates of variability and measure caregiver outcomes including fatigue, anxiety, depression, sleep, self-efficacy, and quality of life at baseline, 4-week intervention completion, and 3 months postintervention.

## Methods

### Phase I

Using a purposive sampling strategy and qualitative descriptive approach, we will identify, recruit, and conduct semistructured key informant interviews with 10 caregivers of children (≤18 years old) with established (≥1 year from diagnosis) FA(s). The purpose of these interviews is to elicit feedback from caregivers that are familiar with the disease and management processes. Caregivers will be informed prior to conducting interviews that their feedback will be used to inform both the content and design elements of FASST as well as the overall utility of the app.

Prior to conducting the interviews, participants will be asked to download and review 2 different free and commercially available smartphone mobile apps: one related to FA management and another related to general lifestyle stress management. Participants will be asked to review both apps for a period of 7-10 days, noting specific elements (content and function) of each app that they would have found helpful during their child’s initial period of FA diagnosis. After 7-10 days, the researchers will schedule one-on-one interviews and ask 8-10 open-ended questions with probes using an interview guide. Questions for the interview will be based on an interview guide; however, flexibility will be afforded to expand upon and probe individual responses. Participants will be asked about their personal experience in the first year of managing their child’s FA, with close questioning regarding the first 4 weeks of management and the perceived impact on their psychosocial well-being and overall quality of life, and to provide user feedback on the acceptability, feasibility, and usability of the 2 mobile apps for the management of FAs and in reducing caregiver burden. Following data analysis, results from the Phase I interviews will be used to inform the design of an mHealth smartphone app (FASST) to meet the specific needs of caregivers of children 18 years of age or younger with a newly diagnosed FA(s) (≤90 days from diagnosis). Caregivers participating in Phase I will receive a US $50 gift card upon study completion.

### Phase II

**Figure 1:**
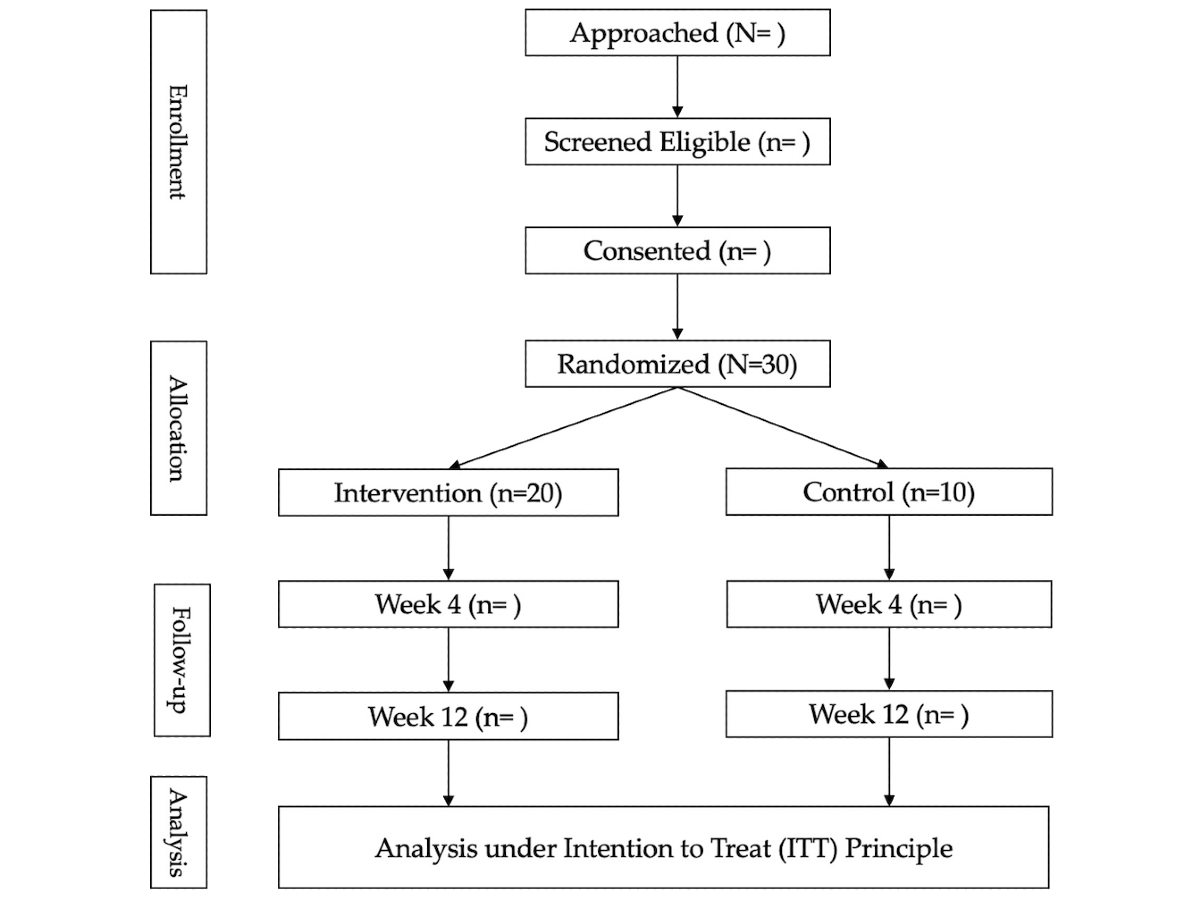
Phase II: CONSORT (Consolidated Standards of Reporting Trials) diagram of participant flow through the study.

The methodology for the Phase II clinical trial follows the directives proposed in the CONSORT (Consolidated Standards of Reporting Trials) 2010 Statement: Extension to randomized pilot and feasibility trials [16]. The overall flow of the Phase II trial is outlined in Figure 1.

#### Design, Setting, and Participants

We designed a longitudinal, randomized controlled trial with 3 study visits (baseline, week 4, and week 16) over a 4-month study period. Study participants will be recruited from an Allergy and Immunology clinic at a large tertiary care academic medical center in South Carolina. Participants from all racial and ethnic backgrounds will be approached and invited to volunteer as research participants. For inclusion in this phase, caregivers must have a child ≤18 years of age who is newly diagnosed (≤90 days from diagnosis) with FA(s). Exclusion criteria include (1) caregivers who carry a diagnosis of cognitive impairment or deficit or an observed lack of understanding of the study demands during the informed consent process and (2) inability to speak or read English. To assess cognitive impairment, when determining eligibility, study personnel will ask potential participants, “Have you been diagnosed by a health care provider with a cognitive impairment or memory disorder?” If the potential participant answers, “yes,” they will not be consented for participation in the study. Eligible and enrolled participants will receive a US $50 gift card for study compensation at each of the 3 data collection points, with participants in key informant interviews (postintervention) receiving an additional US $50 gift card. Study endpoints include successful study completion, participant consent withdrawal, and PI termination due to failure to adhere to the protocol, loss of contact with the participant, or unexpected adverse events.

#### Sample Size

We will recruit 30 caregivers to participate in the intervention phase of the study. Because this is a pilot study and will not be testing hypotheses or proposing use of inferential statistics, a target sample size of 30 is appropriate [17].

#### Randomization and Blinding

We will allocate 20 participants to the study intervention arm and 10 participants to the control arm. The allocation sequence to the 2 groups will be determined using a 2:1 block randomization scheme generated by computer and developed by the study biostatistician. The PI and biostatistician will be blinded to allocation.

#### Intervention

The FASST mHealth intervention will be a multicomponent (3-part) technology-based package delivered via a mobile device and used over the 4-week study period. The intervention will target influences and processes informed by the Individual and Family Self-management Theory [18].

Component 1 (education and support) will consist of continuous access to directed educational resources about FA and its management. These materials will be easily accessible via a mobile device and will include embedded PDFs and links to websites developed and tested by authoritative sources. An example includes education materials provided by Food Allergy Education and Research (FARE), the leading national organization and most trusted source of FA information, programs, and resources, such as the Food Allergy & Anaphylaxis Emergency Care Plan. To address potential literacy barriers, all resources will be provided in a web-compatible format and compliant with current accessibility guidelines.

Component 2 (symptom monitoring and tracking) will consist of a daily symptom log function for tracking overall well-being as well as daily activity; sleep; and psychosocial, emotional, and physical symptoms. All logged symptoms will be uploaded to a portal for assessment by study personnel. The PI or research assistant (RA) will send a daily text reminder to participants randomized to the intervention arm of the study reminding them to complete their daily symptom, activity, and sleep log. Logged symptom trends will be graphically illustrated by the app for participant viewing. Based on feedback from component 2, participants will be given directed guidance, as described in the following paragraphs, related to component 3 (symptom self-management).

Component 3 will consist of symptom-based interventions participants can utilize in real time. For example, if a participant logs symptoms related to anxiety, the app will recommend a brief guided intervention for relaxation, such as meditation or deep breathing. If a participant logs symptoms related to fatigue, the app will recommend the participant listen to a short audio clip that offers ideas for achieving better sleep or suggestions for good sleep hygiene. We will collect data via the app on the frequency and patterns of usage and will also collect measure data at baseline, 4-week intervention completion, and 3 months postintervention. In addition, the RA will send caregivers a weekly text message using a semistructured message script to “check in” with participants and promote engagement.

#### Controls

Participants randomized to the control arm will only receive the caregiver education and support materials (component 1) of the FASST app. All other caregiver baseline and outcome measures will be collected as scheduled by study personnel.

#### Study Procedures

Once consented, participants will be randomized and asked to download the FASST app from the Apple App Store onto their mobile device. Participants randomized to the intervention arm will receive components 1, 2, and 3. Those randomized to the control arm will only receive component 1. The PI or RA will deliver detailed verbal instructions to the participants in both arms of the study on the use of the app and the respective intervention components. Participants will also receive written instructions and a contact number for technical assistance if needed. Baseline measures will be collected during the same meeting. Participants will participate in the intervention over a 4-week period. At the end of the 4-week period, the PI will conduct a postintervention interview via telephone with each individual caregiver participant. The PI will also collect frequency and patterns of usage data from the app portal at 3 months postintervention to assess implementation and preliminary evidence of maintenance. The PI will meet with the RA weekly during the intervention.

#### Postintervention Key Informant Interviews

We will randomly select 5 participants from each study arm in Phase II and ask them to participate in postintervention key informant interviews with the PI to obtain more in-depth data on accessibility, usability, and adherence to intervention. Semistructured interviews will be conducted via phone using a qualitative descriptive approach [19] and will last approximately 30-60 minutes. Questions for the interview will be based on an interview guide; however, the interviewer will allow flexibility with the interview such that subsequent questions may be modified based on participant responses. Participant qualitative interviews will be audio recorded, transcribed, analyzed with directed content analysis, and interpreted to evaluate the feedback.

#### Data Collection and Measures

**Figure 2:**
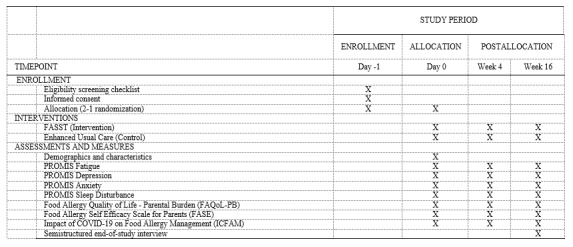
Study SPIRIT diagram. FASST: Food Allergy Symptom Self-Management with Technology for Caregivers; PROMIS: Patient-Reported Outcomes Measurement Information System.

Participants will complete a basic demographic questionnaire at baseline and self-reported measures at baseline, 4-week intervention completion, and 3 months postintervention. Participants will complete the questionnaire and measures using their personal mobile device or computer. The SPIRIT diagram (Figure 2) summarizes the schedule of enrollment, interventions, and assessments across the study.

### Data Management

This study will use Research Electronic Data Capture (REDCap) for e-consenting, data capture, and data management. REDCap is a software toolset and workflow methodology for the electronic collection and management of research and clinical trials data. REDCap provides secure, web-based, flexible applications, including real-time validation rules with automated data type and range checks at the time of data entry. Exports are made available for several statistical packages including SPSS, SAS, SATA, R, and Microsoft Excel.

### Data Analysis

We will collect data from multiple measures informed by the RE-AIM framework to assess feasibility and inform future efficacy and effectiveness trials. Variables will include those pertaining to the study procedures and processes as well as participant demographic and clinical variables. Specifically, 95% confidence intervals for proportions will be used to estimate dichotomous outcomes including the proportion of caregivers who agree to participate out of the number approached and proportions providing daily symptom monitoring or tracking, using the education component, etc. For the continuous feasibility measures (patient satisfaction scores from patient surveys and end-of-study interview), frequency distributions and the median and mean responses (with 95% confidence intervals) will be obtained.

Demographic and clinical variables obtained at baseline will be described via measures of central tendency (mean, median), variability, and frequency distributions, as appropriate. In addition, demographic and clinical characteristics for those who completed the study protocol versus those who did not complete the study protocol will be compared to better describe the population for this study. For continuous measures for the caregiver (fatigue, anxiety, depression, quality of life, sleep disturbance, and self-efficacy), the difference between pre- and postintervention measurements will be estimated via 95% confidence intervals.

Data collected from postintervention key informant interviews will be analyzed using directed content analysis [20] and nVivo qualitative data analysis software version 12 [21]. Consistent with the directed content analysis approach, initial coding categories will be identified according to the guiding theoretical model and for this study, will reflect the RE-AIM domains.

### Data Safety and Monitoring

This study employs the use of a National Institutes of Health (NIH)/National Institute of Nursing Research (NINR) Scientific Review Group–approved Data and Safety Monitoring Plan. The Data and Safety Monitoring Plan utilizes a Safety Monitoring Committee comprised of an independent nurse practitioner (registered nurse, PhD), the study biostatistician (PhD), and the trial director (MS). After the initial participant study enrollment, the Safety Monitoring Committee is set to convene semiannually to review all adverse events, monitor the study safety profile, and make recommendations regarding study modification, termination, and continuance.

### Ethical Considerations

The study was approved by an institutional review board, conforms to the Declaration of Helsinki, and will be conducted in compliance with Good Clinical Practices of the International Conference of Harmonization. Parental caregivers provide written informed consent for their study participation. The study was registered on 08/14/2020 in ClinicalTrials.gov (NCT04512924).

### Resource Sharing

Data collected from the PROMIS (Patient-Reported Outcomes Measurement Information System) measures will be entered for resource sharing through the Biomedical Research Informatics Computing System at the NINR. The data obtained in the current study will be available from the PI upon reasonable request after publication of the results on the main research questions. Results will be shared with study participants and made publicly available in compliance with NIH Open Access Policy.

## Results

### Progress to Date

**Figure 3:**
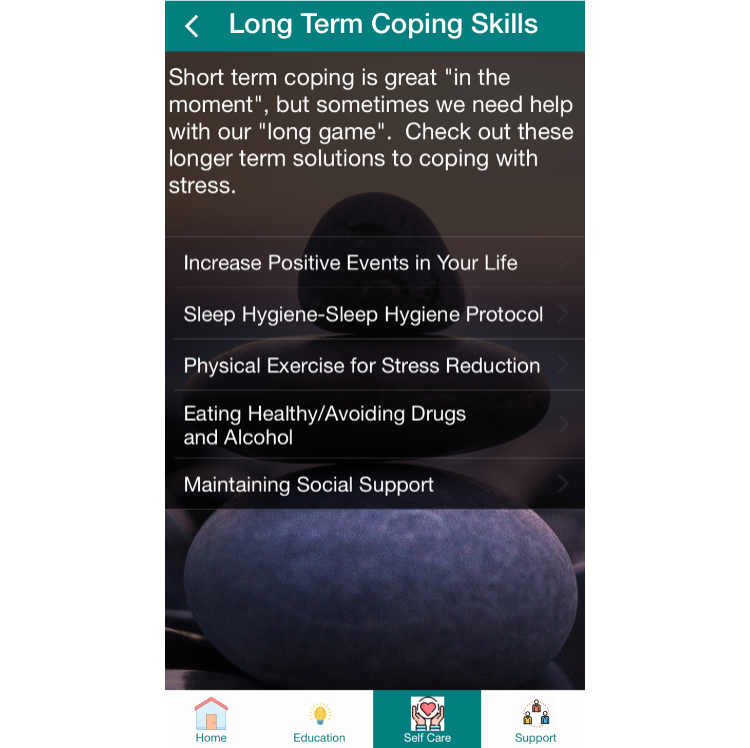
Sample Food Allergy Symptom Self-Management with Technology for Caregivers (FASST) mobile health (mHealth) smartphone app screen image.

**Figure 4:**
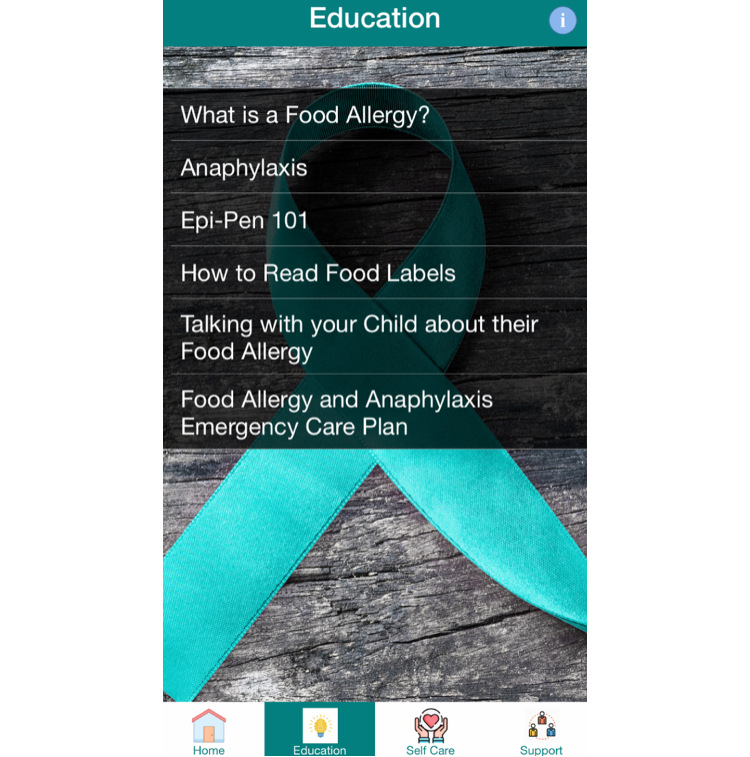
Sample Food Allergy Symptom Self-Management with Technology for Caregivers (FASST) mobile health (mHealth) smartphone app screen image.

All Phase I study activities have now been completed. Based upon this work, we are presently working with our software development team to make final refinements to the FASST app prior to Phase II implementation. In conjunction with our software development team, the app is being developed for the iOS platform. Sample smartphone screen images from the mHealth app are provided for illustrative purposes in Figures 3 and 4. Phase II study enrollment is expected to commence in 2021.

### Lessons Learned

During the formative Phase I part of this study, multiple lessons were learned, mainly pertaining to needed operational and procedural changes so as to ensure the continuity of human subject research during the COVID-19 pandemic.

Recruitment for Phase I began in late April 2020, just as community and institutional social and physical distancing restrictions related to COVID-19 were mandated, and as such, the original protocol was modified through an amendment to allow for completion of all study procedures remotely (eg, participant interviews conducted via phone instead of face-to-face and study compensation mailed to participant home address rather than in person). All phone interviews were successfully audio-recorded and transcribed for analysis; however, this mode of contact with participants limited capture of nonverbal cues that may have led to further probing. Another Phase I challenge involved postponed development of the FASST mobile app. Our technology development team was presented with higher priority institutional demands in response to COVID-19. As such, progress on app development experienced significant delays.

While COVID-19 has presented several challenges to the study, we believe it also has resulted in some unintended consequences that benefited the timely completion of Phase I study activities. During this phase, 11 potentially eligible participants were identified through their electronic health records and approached by the researchers regarding study interest and participation. All participants (11/11) responded within 24-48 hours of initial contact and verbally expressed both an interest and a willingness to participate in Phase I. Of these interested participants, 91% (10/11) were screened eligible, and 100% (10/10) successfully completed all Phase I activities. We hypothesize these observed high rates were possibly related to the participants’ limited physical mobility and lack of social contact with others outside of the home environment due to imposed COVID-19 restrictions at the time (eg, stay-at-home mandates, school and church closures, and modified work routines) that in turn improved our accessibility to research participants and increased their willingness to participate in this research.

## Discussion

Documentation of psychosocial manifestations among caregivers of children newly diagnosed with FA is becoming more prevalent in the literature. However, interventions to address such manifestations have largely gone unrecognized within the medical community. Further, studies of technology-based interventions that focus on symptom self-management in this population are absent from current literature. Application of mHealth to a variety of chronic health conditions has demonstrated positive associations with self-management outcomes [22]. The present study will contribute to the body of scientific knowledge by creating, delivering, and evaluating an mHealth, technology-based, scalable, self-management strategy to address the potential physical, emotional, and psychosocial outcomes experienced by caregivers of children newly diagnosed with an FA. By focusing on psychosocial symptoms, such as fatigue, our work will address an unmet need recognized within the literature but neglected by providers. Our scalable strategy to engage caregivers during a critical time period in the caregiving/condition trajectory (≤90 days from diagnosis) when psychosocial functioning is at high risk may also reveal other psychosocial manifestations not previously recognized in this specific population. We feel the novel approach of this project has the potential to demonstrate both cost-effectiveness and sustainability. In addition, our work may address barriers to accessing care such as transportation, work schedules, and childcare through the application of technology. Addressing these barriers lends to the translatability of the intervention to other populations of caregivers of children with chronic health conditions.

## Abbreviations

CONSORT: Consolidated Standards of Reporting Trials
FA: food allergy
FARE: Food Allergy Education and Research
FASST: Food Allergy Symptom Self-Management with Technology for Caregivers
FIA: food-induced analaphylaxis
mHealth: mobile health
NIH: National Institutes of Health
NINR: National Institute of Nursing Research
PI: principal investigator
PROMIS: Patient-Reported Outcomes Measurement Information System
RA: research assistant
RE-AIM: Reach, Effectiveness, Adoption, Implementation, and Maintenance
REDCap: Research Electronic Data Capture
